# Intra- and Inter-Task Reliability of Spatial Attention Measures in Pseudoneglect

**DOI:** 10.1371/journal.pone.0138379

**Published:** 2015-09-17

**Authors:** Gemma Learmonth, Aodhan Gallagher, Jamie Gibson, Gregor Thut, Monika Harvey

**Affiliations:** 1 Centre for Cognitive Neuroimaging, Institute of Neuroscience and Psychology, University of Glasgow, Glasgow, United Kingdom; 2 School of Psychology, University of Glasgow, Glasgow, United Kingdom; University of Nottingham, UNITED KINGDOM

## Abstract

Healthy young adults display a leftward asymmetry of spatial attention (“pseudoneglect”) that has been measured with a wide range of different tasks. Yet at present there is a lack of systematic evidence that the tasks commonly used in research today are i) stable measures over time and ii) provide similar measures of spatial bias. Fifty right-handed young adults were tested on five tasks (*manual line bisection*, *landmark*, *greyscales*, *gratingscales* and *lateralised visual detection*) on two different days. All five tasks were found to be stable measures of bias over the two testing sessions, indicating that each is a reliable measure in itself. Surprisingly, no strongly significant inter-task correlations were found. However, principal component analysis revealed left-right asymmetries to be subdivided in 4 main components, namely asymmetries in size judgements (*manual line bisection* and *landmark*), luminance judgements (*greyscales*), stimulus detection (*lateralised visual detection*) and judgements of global/local features (*manual line bisection* and *grating scales*). The results align with recent research on hemispatial neglect which conceptualises the condition as multi-component rather than a single pathological deficit of spatial attention. We conclude that spatial biases in judgment of visual stimulus features in healthy adults (e.g., pseudoneglect) is also a multi-component phenomenon that may be captured by variations in task demands which engage task-dependent patterns of activation within the attention network.

## Introduction

Spatial attention asymmetries are well documented in the general population. Typically, healthy adults display a processing advantage towards the left side of space (“pseudoneglect”), likely as a result of right cerebral hemisphere dominance for spatial attention [1]. This left-sided advantage manifests as a systematic overestimation of the magnitude of target features that are located on the left and has been observed across various experimental tasks. Yet as a result of these multiple methods of testing, a variety of task demands are introduced. It remains unclear whether the most frequently used tasks in the spatial attention literature capture a bias that is representative of a single, common underlying aspect of spatial attention bias or whether pseudoneglect is instead a multi-component phenomenon.

In the clinical setting, patients with hemispatial neglect are known to exhibit dissociations of performance across subtests of batteries that are intended to assess spatial attention deficits, e.g the Behavioural Inattention Test [2–7]. These subtest-specific deficits are generally correlated with the location of the lesion within the brain. Verdon et al., [8] reported that damage to the right inferior parietal lobe negatively affects the ability to direct attention to the ipsilesional hemispace on tasks involving general visuospatial perception components (i.e. the line bisection task and text reading). Damage more anteriorly within the right dorsolateral prefrontal cortex impairs visuomotor exploration (i.e. object cancellation tasks), whilst patients with temporal lobe damage perform less well for object-centred (“allocentric”) perception (i.e. the Ota task). There is therefore strong evidence that hemispatial neglect is a multi-component disorder involving disruption to distinct aspects of the attentional network.

We reason that, analogous to the dissociations within the neglect patient population, the assorted tasks used to measure attentional bias in the healthy population represent a potential method of partitioning out pseudoneglect into distinct components. The lack of relationship between various spatial attention tasks was first documented by Luh [9] twenty years ago, who reported no significant correlation between spatial asymmetries elicited in response to chimeric face judgements, dot-filled rectangles, Muller-Lyer shapes and manual line bisection performance in non-lesioned adults. Similarly, Nicholls, Bradshaw & Mattingley [10] presented tasks requiring a judgement of size (the *shape* task), number (the *stars* task) and shading gradients (the *greyscales* task) and although bias measures were found to be highly consistent *within* each individual task (as indexed by split-half reliability), there was little evidence of a consistent relationship *between* the 3 tasks. This suggests that a dissociation exists between tasks which involve different cognitive demands similar to the observations in hemispatial neglect patients. We aimed to evaluate both intra- and inter-task reliability in pseudoneglect explicitly, with a focus on tasks used in the current spatial attention literature.

**Fig 1:**
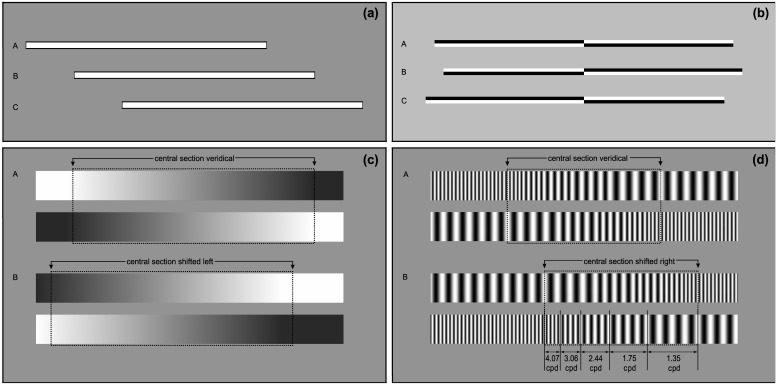
Examples of the (a) manual line bisection (MLB), (b) landmark (LM), (c) greyscales (GRE) and (d) gratingscales (GRA) stimuli. **1a)** Stimulus B is centred at the horizontal midpoint of the screen. Stimuli A and C represent the most extremely deviated stimuli along the horizontal axis, with Stimulus A jittered 160 pixels (3.8° visual angle (VA)) leftward and C jittered 160 pixels rightward relative to centre. **1b)** The left and right sides of Stimulus A are of equal length, the left side of B is shorter by 24 pixels (0.53°) and the right side of C shorter by 24 pixels. **1c)** A central “zone of interest” (640 pixels wide, 15.1°) was shifted in 10-pixel (0.24°) increments leftward and rightward. The shading gradient is continuous from left to right in Stimulus A and the central section is shifted leftward by -80 pixels (1.9°) in B. **1d)** A 400-pixel wide (9.47°) zone of interest was shifted in 12-pixel (0.29°) increments leftward and rightward. The zone is centred in Stimulus A and shifted rightward by 96 pixels (2.28°) in B.

One of the most commonly-used tests of hemispatial neglect and pseudoneglect, the manual line bisection (MLB) task, requires participants to place a mark at the midpoint of a horizontal line (Fig 1a). The deviation of this mark relative to the true midpoint determines the direction and extent of attentional asymmetry, which is typically deviated rightward in patients with neglect and leftward (although to a lesser magnitude) in the healthy population. The traditional paper-and-pencil version of the MLB task involves the co-ordination of both visuospatial and motor abilities [9] and replicating these demands on a computer screen can prove problematic, possibly inducing a rightward bias that may be due to the presentation being delivered in extrapersonal, rather than peripersonal, space [11]. Many studies have also reduced the motor demands of the MLB task by presenting a cursor at one end of the horizontal line, with instructions to incrementally move this towards the midpoint using keyboard buttons [11– 14]. Recent studies have incorporated the computer mouse pointer to more closely replicate the motor action that is required in the paper versions [15–18].

Performance variability has been reported in the MLB task, both within and between individuals [19–20] and the direction of the pseudoneglect bias appears to be partially dependent on the spatial location of the line along the horizontal plane (see [20], for review). There is however mixed consensus on the direction of this “positional uncertainty” effect, with some studies finding a bidirectional, centrifugal shift of bias leftward (or rightward) as the line is jittered further into the left (or right) side of space respectively [21– 23]. Others have shown a consistent leftward bias when stimuli are positioned in both left and right hemispace, with performance more extremely leftward-deviated when presented to the left [9].

A non-manual variant of the MLB task—the *landmark* task (LM), (also called “tachistoscopic line bisection” [24])—is a common alternative measure of spatial attention bias which also serves to reduce the requirement for complex manual (pointing) movements [22, 25–27] (Fig 1b). Subjects are instructed to make a two-alternative forced choice decision regarding the length of two halves of a pre-bisected line. Healthy young adults demonstrate a systematic leftward bias of the subjective midpoint of the landmark lines, that is consistent with an overestimation of the size of left hemispace [28–34]. Given the similarity between the MLB and LM tasks, it is perhaps unsurprising that a consistent correlation in the direction and magnitude of pseudoneglect bias has been observed between the two tasks in healthy adults ([9, 11, 18, 22], but see also [35]). In support of this, functional magnetic resonance imaging (fMRI) during LM and LB performance has indicated a similar pattern of right cerebral hemisphere recruitment for both tasks (right dorsal fronto-parietal network activation for both tasks [35] with additional involvement of the right frontal eye fields for LB) [12].

Although the MLB and LM tasks require a judgement of the relative *size* of a stimulus presented within the left and right hemispace, pseudoneglect has also been demonstrated in tasks involving a range of target features. The *greyscales* task (GRE) requires a comparative luminance (“darkness”) judgement to be made between two parallel horizontal lines containing a mirror-imaged linear contrast gradients (Fig 1c). The bar in which the dark section is located on the left side of space tends to be perceived as darker overall, compared to when it is placed on the right [10, 36–41]. Most importantly, the left side is reported darker even when the bars are equiluminant, indicating a perceptual overestimation of the darkness of the left side of the stimulus. Nonetheless, there is a large reported variation of bias in this task, with some individuals displaying a clear leftward bias and others rightward [37, 42–43], although performance does appear to be reliable over multiple testing days which may indicate the presence of distinct population subgroups for the GRE task [43]. In addition, in a first attempt at cross-task comparisons, contrasting the bias observed in GRE with other tasks of spatial bias, Mattingley et al., [37] and Nicholls & Roberts [44] found only weak correlations with LB bias, which may be related to the motor demands required for LB but not GRE task performance. Additionally, Heber et al., [45] reported no correlation between the GRE and LM tasks, further indicating that size judgements (in response to MLB and LM tasks) and luminance judgements (GRE) give rise to distinct and separable spatial attention asymmetry effects.

The *gratingscales* task (GRA) is derived from the GRE task and exploits the observation that size and spatial frequency perception share common features [46–48]. The GRA also involves the presentation of two mirror-imaged parallel horizontal rectangles, but comprising sine-wave gratings of increasing/decreasing spatial frequency (Fig 1d). When instructed to indicate the bar containing more “thin stripes” (i.e. high frequency sine-wave gratings), subjects are more likely to indicate the bar with the target feature located on the left side of space [48–51]. However, when instructed to judge the “thick stripes” (i.e. low frequency gratings) instead there is a “cross-over” of bias where the *right* side is favoured. Nonetheless, this rightward bias is comparatively smaller in magnitude, alluding to an overall right hemisphere dominance that is similar to the other tasks described [51–52]. Because this cross-over effect is positively correlated within individuals it appears to be indicative of a stable set of distinct attentional mechanisms that are responsible for the processing of high and low spatial frequencies [51]. Importantly, the GRA and GRE tasks are positively correlated measures of pseudoneglect when the stimuli are presented for a short duration (240 or 500ms) but not at longer presentation times [48].

Finally, the *lateralised visual detection* (LVD) task as described by Hilgetag et al., [53] is intended to provide a simple measure of sustained attention during which participants detect small dots that appear very briefly in the left and right sides of space. Stimuli sizes are typically titrated to an individual’s peri-threshold (50%) accuracy to equate the difficulty of the experiment across individuals [17, 53–57]. Using this paradigm, Hilgetag et al., [53] and Thut et al. [57] found greater detection accuracy for left- compared to right-presented stimuli, that is consistent with pseudoneglect. We have also recently reported a stable discrimination sensitivity (*d-prime*) bias towards the left hemispace for this titrated task over multiple testing days in young adults (ages: 18–25), although this effect was less consistent in older adults aged over 60 [55].

Here we report a correlational study where these five tasks that have been commonly used to measure spatial attention asymmetries were completed over two testing sessions on different days. We aimed to investigate whether i) the direction and magnitude of bias is correlated within tasks across the two days (i.e. *intra*-task reliability) and ii) if the bias is correlated between each of the five tasks (i.e. *inter*-task reliability).

We aimed to update the inter-correlational studies of Luh [9] and Nicholls et al [10] by using tasks that are commonly used to measure spatial attention biases in the current literature. Although these previous studies found very little between-task reliability, it was hoped that a more consistent relationship would be observed amongst these more recently-used tasks. Conversely, if our results indicated a continued lack of equivalence between spatial attention tasks, we would conclude that pseudoneglect is indeed multi-component and task-dependent and an assumption of equivalence thus counterproductive to the investigation of the neural underpinnings of pseudoneglect and its functional implications.

## Methods

### Participants

Fifty adults were recruited (35 females, mean age = 22.56 years; SD = 4.46, range = 18–38) and a further 1 participant was excluded due to failure to complete the second session. All were right-handed and had normal or corrected-to-normal vision. The study was approved by the University of Glasgow College of Science and Engineering ethics committee and written, informed consent was obtained from each participant.

### Procedure

Testing took place over two sessions (at least 24 hours apart) in a repeated-measures design. At the start and end of each session participants indicated their subjective alertness on a linear scale (0 = almost asleep, 100 = fully alert). They were seated in front of a computer screen with their midsagittal plane aligned with the screen. Five blocks were presented during each testing session (1 block per task) in an order that was counterbalanced across participants. The 5 tasks were i) *landmark* (LM), ii) *manual line bisection* (MLB), iii) *greyscales* (GRE), iv) *gratingscales* (GRA) and v) *lateralised visual detection* (LVD) and the sequence of presentation was the same on Day 1 and Day 2 per participant to control for possible task-order effects. A short practice block (approximately 20 trials) preceded each task and participants were instructed to take a short break between blocks if required.

### Stimuli

Stimuli were presented with E-Prime 2.0 (Psychology Software Tools Inc., Pittsburgh, PA) using a Dell Precision 380 PC and a 19” Dell 1908FP UltraSharp LCD flat screen monitor with a 1280x1024 pixel resolution. One pixel measured approximately 0.29mm^2^. The viewing distance was fixed with a chin rest at 0.7m.

#### Manual line bisection task (MLB)

The manual line bisection task was designed to closely replicate the paper-and-pencil version on a computer screen. Horizontal white lines (805 x 15 pixels) (approximately 23.5cm x 0.4cm; 19.06 x 0.33° visual angle (VA)) were presented on a grey background in the centre of the screen (Fig 1a). The outermost 2 pixels bordering the line were shaded black. The line was jittered at 9 positions along the horizontal axis on a trial-by-trial basis (0 = centred, and 40, 80, 120 and 160 pixels (0.95, 1.9, 2.85 and 3.8° VA) to the left and to the right of veridical centre). The mouse pointer was set to appear at the same starting location in the upper midpoint of the screen at the start of each trial (screen co-ordinates: X = 640, Y = 40 pixels; 11.17° above fixation). Participants were instructed to move the mouse pointer down towards the line and, using their right index finger, left-click on the horizontal midpoint of the line as accurately as possible. They were informed that the vertical co-ordinate did not matter. The line remained on the screen until a response was made, or 6 seconds elapsed without a response. The stimulus of the next trial appeared 1000ms thereafter (with the mouse pointer reset at the starting location). A total of 108 trials were presented (9 line positions repeated 12 times).

#### Landmark task (LM)

The LM task was adapted from McCourt [32] and Milner, Brechmann & Pagliarini [22] (see also [27–30]). A centred fixation cross (15x15 pixels; 0.58° VA) appeared for 1000ms followed by a stimulus for 150ms. The fixation cross then reappeared until a response was given. Subjects indicated whether the left or right side of the line was shorter by keyboard response using their right hand. Stimuli consisted of horizontal 100% Michelson contrast lines measuring 800x14 pixels (approximately 23.5cm x 0.4cm; 19.06 x 0.33° VA) (Fig 1b). Each line was vertically transected at the veridical centre of the screen (i.e. at the same position as the fixation cross) but the length of the left and right sections varied across trials. The most asymmetrical (left side vs right side) stimuli differed by 24 pixels (0.53°) and the asymmetry reduced in 3-pixel (0.07°) increments until the two sides were of equal length. Thus, 17 stimuli of varying asymmetry were created. The landmark block consisted of 136 trials (17 stimuli repeated 8 times (x4 where the upper left and lower right sections were shaded black (e.g. Stimuli A and C) and x4 where the upper left and lower right were white (e.g. Stimulus B)).

#### Greyscales task (GRE)

The greyscales task was adapted from Mattingley et al., [36–37] and involved the presentation of two parallel horizontal rectangles, one above the other (Fig 1c). The rectangles were shaded along a smooth luminance gradient so that one end of the rectangle was fully black and the other end fully white. The rectangles were mirror-images along the horizontal and vertical axes, so that half of the trials involved upper bars that increased in luminance from left-right, with the lower bar increasing from right-left. The remaining trials contained the opposite configuration (top: right-left gradient, bottom: left-right). A centred fixation cross (15 x 15 pixels; 0.58° VA) appeared for 1000ms followed by a stimulus for 150ms. The fixation then reappeared, during which participants indicated whether the top or bottom bar was darker overall using the “up” or “down” keyboard arrows. The next trial began when a response was given. Each rectangle measured 800 x 100 pixels (approximately 23.5cm x 2.9cm; 19.06 x 2.37° VA), with 41 pixels (0.97°) between the two bars. As per Niemeier, Stojanoski & Greco [48] the task was modified to allow an estimation of spatial bias with psychometric functions. A central “zone of interest” section comprising 640 pixels (80% of the total length, 15.1°) was shifted in 10-pixel (0.24°) increments to the left or to the right to provide 17 different stimuli. The most extremely asymmetric rectangles differed by 80 pixels (1.9°; -10% or +10% of total length). The remainder of the bar was then filled in with solid black/white. Thus, Fig 1c Stimulus B shows the central section shifted leftwards and the participant would be likely to perceive the lower bar to be darker overall. One block comprised 136 trials (17 stimuli repeated 8 times (x4 where the upper left and lower right sections were shaded white (e.g. Stimulus A) and x4 where the upper left and lower right were black (e.g. Stimulus B)).

#### Gratingscales task (GRA)

The gratingscales task was adapted from Niemeier, Stojanoski & Greco [48]. Similar to the GRE task, two mirror-imaged, parallel horizontal bars (800 x 100 pixels: approximately 23.5cm x 2.9cm; 19.06 x 2.37° VA) were presented, but instead of a shaded gradient the stimuli contained sine-wave gratings (Fig 1d). The grating was high-frequency (“HiSF”) at one end of the rectangle (35 pixels per cycle; 1.35 cycles per degree of visual angle (cpd)) and low-frequency (“LoSF”) at the opposite end (11 pixels per cycle; 4.07 cpd). A central “zone of interest” measuring 400 pixels (50% of the total length, 9.47°) was shifted in 12-pixel (0.29°) increments to the left or to the right to provide 17 different gratingscales stimuli. The most extremely asymmetrical rectangles differed by 96 pixels (2.28°; -12% or +12% of total length) (Fig 1d, Stimulus B). The remainder of the line was then filled in with continuous HiSF and LoSF gratings. The zone of interest contained sine waves of 5 different spatial frequencies, with 4 sine wave cycles per frequency, which ranged from LoSF = 35 pixels per cycle, through 26, 19, 14 and the highest frequency of 10 pixels per cycle (i.e. the number of pixels per cycle reduced by a factor of approximately x 0.74). A centred fixation cross (15x15 pixels; 0.58°) appeared for 1000ms followed by a stimulus for 150ms. The fixation then reappeared, during which participants indicated whether the top or bottom line had more “thin stripes” (i.e. high frequency gratings) overall using the “up” or “down” keyboard arrows. Fig 1d Stimulus B shows the central section shifted rightward and the participant would be likely to perceive the lower bar as containing more thin stripes overall. One block comprised 136 trials (17 stimuli repeated 8 times (x4 where the upper left and lower right sections were HiSF (e.g. Stimulus A) and x4 where the upper left and lower right were LoSF (e.g. Stimulus B)).

#### Lateralised visual detection task (LVD)

The task was adapted from Hilgetag et al., [53] and was similar to the task used in [17, 54–57]. Stimuli consisted of small black squares or rectangles (with the longer edge along the horizontal axis) presented against a grey screen (luminance = 179, hue = 160). The squares were of 5 different sizes (1x2, 2x2, 2x3, 3x3 and 3x4 pixels; between 0.024 x 0.047° and 0.87 x 1.16° VA) and were presented either to the left (-145mm; -16.5°), or to the right (+145mm; +16.5°) of fixation (no placeholders presented). One block comprised 132 trials (12 left and 12 right for each of the 5 stimulus sizes, plus 12 blank “catch” trials where the screen remained blank). Participants used their right hand to indicate on a keyboard when the stimulus appeared on the left (index finger) or right (middle finger) and they were instructed to withhold their response when no stimulus was detected. A centred fixation cross (15x15 pixels; 0.58°) appeared for 1000ms followed by a stimulus for 40ms. A blank response screen then appeared for a fixed duration of 1750ms (to accommodate false negatives and catch trials), after which a new trial began.

### Analysis

#### LM, GRE and GRA tasks

The LM, GRE and GRA tasks were analysed using the same method to ensure comparability of results. Accuracy for each of the 17 stimulus asymmetries was converted into a percentage of trials where the subject perceived the stimulus to be either shorter (LM) / darker (GRE) / have more “thin stripes” (GRA) on the *left* side of space. Psychometric functions were then fitted to the data for each individual and the point of subjective equality (PSE) for each task was obtained using the cumulative logistic function described by the equation:

$$f(\mu ,\;x,\;s)\;=\;1/(1+\text{exp}\left(\frac{x-\mu }{s}\right))$$

Where *μ* is the point on the x-axis that corresponds to 50% left and 50% right-response rate, *x* represents the transector locations and *s* is the psychometric curve width. Curve widths provide a measure of task ability, with a narrow curve width indicative of good performance [58–59].

#### MLB task

The x- and y-pixel co-ordinates of the screen that was clicked using the mouse were logged by E-Prime. This subjective x-co-ordinate was subtracted from the co-ordinate of the true midpoint location and the mean bias and standard deviation were calculated for each individual. Responses that were greater than 2.5 SD above and below the individual’s mean were excluded due to a few extreme values (151 trials = 1.42%) made in error, whilst moving the mouse towards the stimulus from the starting position. The adjusted mean was then recalculated which provided an overall bias score (in pixels) towards either the left (negative value) or the right side (positive value) for the manual line bisection block.

#### LVD task

Two methods of analysis were used to calculate spatial attention bias in the LVD task due to different approaches used in previous studies.

**1. D-prime (*d’*):** As per Learmonth et al., [55] this method uses visual detection sensitivity and takes into account both percentage accuracy for each side of space (“hits” when stimuli are present and “false alarms” in response to catch trials)[60–61]. *D*’ was calculated using the function:

$$d^{'}\;=\;z(Hits)-z(FalseAlarms)$$

[62] where *z* represents the z-score for each side of space. Larger *d*’ scores represent a greater sensitivity for detecting stimuli relative to false positives. A *d*’ lateralisation index was then calculated by subtracting Left visual field (VF) *d*’ from Right VF *d*’.

**2. Psychometric function fitting (PF 50%):** Another method of analysis, bringing the method of analysis into alignment with the LM, GRE and GRA tasks reported here, is to fit psychometric functions for percentage accuracy on the 5 stimulus sizes. Individual curves were fitted separately for left- and right-presented stimuli and PSEs and curve widths were extracted. To fit the curves, the 5 stimulus sizes were labelled as 1 = 1x2 pixels, 2 = 2x2, 3 = 2x3, 4 = 3x3 and 5 = 3x4 and therefore a PSE of 1.5 indicates that the participant reached 50% accuracy (PF 50%) at a stimulus size that lies half way between 1x2 and 2x2 pixels. A small PF 50% value represents better performance compared to larger PF 50% values (i.e. 50% accuracy achieved at a smaller pixel size). A measure of lateralised spatial bias was then calculated by subtracting the Right VF PSE from the Left VF PSE. The PF 50% and *d*’ methods were found to be strongly correlated on both testing days (Day 1: r = 0.884, p<0.001; Day 2: r = 0.965, p<0.001; Mean Days 1+2: r = 0.937, p<0.001).

## Results

### Counterbalancing

A Friedman test confirmed that the tasks were adequately randomised in terms of presentation order (χ^2^(4) = 1.232, p = 0.873). Mean ranks *MLB*: 2.96 (SD = 1.32), *LM*: 3.02 (SD = 1.39), *GRE*: 3.04 (SD = 1.48), *GRA*: 3.16 (SD = 1.53), *LVD*: 2.82 (SD = 1.38).

### Subjective alertness

A 2x2 analysis of variance (ANOVA) (TIME: *pre-* vs *post-experiment* x DAY: Day *1* vs *Day 2*) on subjective alertness scores found that alertness generally reduced over the course of the experiment [Mean *pre* = 74.75, SD = 11.46, Mean *post* = 64.9, SD = 15.29; main effect of TIME: F(1,49) = 25.51, p<0.001] but did not differ between the two testing days. No other effects proved significant.

### Task performance

While spatial bias is our primary measure of interest, it does not allow us to gauge the precision of the participants’ performance, i.e. to what extent participants were actively engaged with the task. We therefore first analysed curve width, a measure of the precision of the subjective midpoint judgement (for each block), available for all tasks employed (LM, GRE, GRA and LVD), except MLB (no psychometric functions fitted). A steep slope indicates high task precision, whereas a shallower curve is obtained when the individual is less precise [58–59]. Each task showed a slight mean reduction of curve width on the second day relative to the first, reflecting a slight improvement in precision gained by task learning, but paired samples t-tests indicated that the reduction was not significant for the LM, GRA and LVD (all p-values ≥0.149). Only the mean GRE curve was significantly narrower on Day 2 compared to Day 1 [t(49) = -2.424, p = 0.019]. For the LM, GRE and GRA tasks, the intra-task curve widths were correlated between Day 1 and Day 2 across participants (Pearson’s r: LM r = 0.312, p = 0.028; GRE r = 0.546, p<0.001; GRA r = 0.67, p<0.001) indicating good performance consistency over the two days. Likewise, for the LVD task, curve widths obtained from curve fitting (analysis of PF 50%) were correlated across the two testing days for each visual field (Day 1 vs Day 2, Left-presented stimuli: r = 0.335, p = 0.018; Right-presented stimuli: r = 0.380, p = 0.007). An average curve width was calculated across days (Day 1, Day 2) and the curve widths for the Left versus Right stimuli were significantly correlated (r = 0.452, p = 0.001) indicating that participants performed the task with similar precision across both days and in both sides of space. Overall, this indicates significant performance consistency, suggesting that spatial bias values across days and tasks are interpretable (are not contaminated by poor task engagement across days or tasks).

### Spatial bias per task

#### MLB task

**Fig 2:**
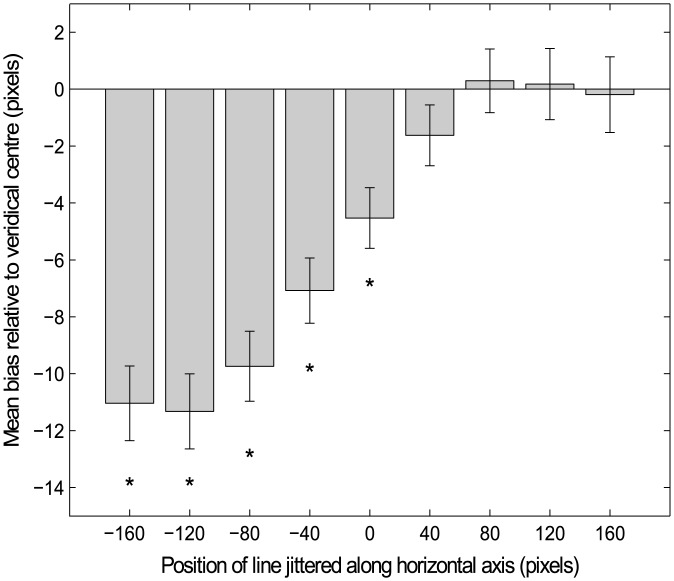
Mean bias for the MLB task. * represents a significant leftward bias (p<0.002).

The group-averaged MLB bias was first analysed for each of the 9 jittered line positions along the horizontal axis (averaged across the two testing days in Fig 2). Separate one-sample t-tests against zero for each of the 9 positions confirmed a bias towards the left of the true horizontal centre when the line was positioned at the veridical centre and when jittered towards the left of the screen (position *0 veridical*: [t(49) = -3.533, p = 0.001]; *-40 left*: [t(49) = -4.926, p<0.001]; *-80 left*: [t(49) = -6.286, p<0.001] *-120 left*: [t(49) = -6.589, p<0.001] and *-160 left*: [t(49) = -6.455, p<0.001]) but participants did not err significantly when the line was jittered towards the right (positions *+40*, *+80*, *+120* and *+140 right*). A repeated measures ANOVA for the 9 line positions revealed a main effect of POSITION [F(1,49) = 39.77, p<0.001, ηp^2^ = 0.448]. Paired samples t-tests found no difference in bias between the two most extreme leftward lines (*-160 left* and *-120 left* of centre) but there was a significant incremental leftward shift between positions *0* vs *-40* [t(49) = -3.029, p = 0.004], -*40* vs *-80* [t(49) = -3.897, p<0.001], both significant at corrected α = 0.00625, and *-80* vs *-120* [t(49) = -2.526, p = 0.015] indicating an increase in leftward bias magnitude as the line was shifted further into left hemispace and confirming the findings of Luh et al., [9]. A compound measure of bias in this task (collapsing across all 9 line positions and the two days) revealed a consistent leftward bias (one sample t-test against zero: t(49) = -3.85, p<0.001).

#### LM, GRE and GRA tasks

**Fig 3:**
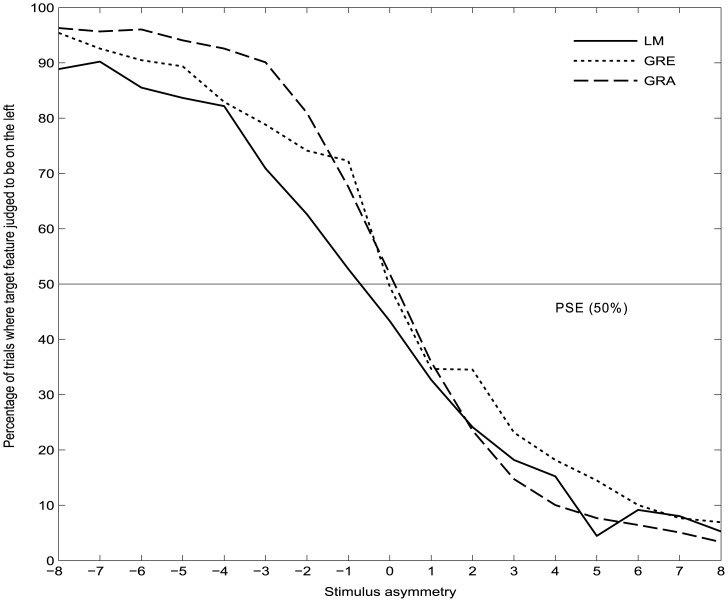
Mean psychometric function curves for the LM, GRE and GRA tasks. The asymmetry of the presented stimulus is shown on the x-axis, where 0 = “both sides equal length” (LM) or “both bars equal darkness/thin stripes” (GRE/GRA respectively). Negative asymmetry values represent trials where the target feature is located on the left side and positive values on the right side. One unit on the x-axis equates to 3 pixels (0.07°) for the LM task, 10 pixels (0.24°) for GRE and 12 pixels (0.29°) for GRA.

The group-averaged (mean of Day 1 and Day 2) psychometric function curves used to calculate the point of subjective equality (PSE) and curve widths for the LM, GRE and GRA tasks are shown in Fig 3. Analysis of PSEs (PF 50%) per task (collapsed across days) revealed a consistent leftward bias only for the LM task (one sample t-test against zero: t(49) = -3.47, p = 0.001). PSEs of the GRE and GRA tasks were not significantly different from zero (GRE [t(49) = 1.52, p = 0.136], GRA [t(49) = 1.136, p = 0.25], data collapsed over the two days).

#### LVD task

**Fig 4:**
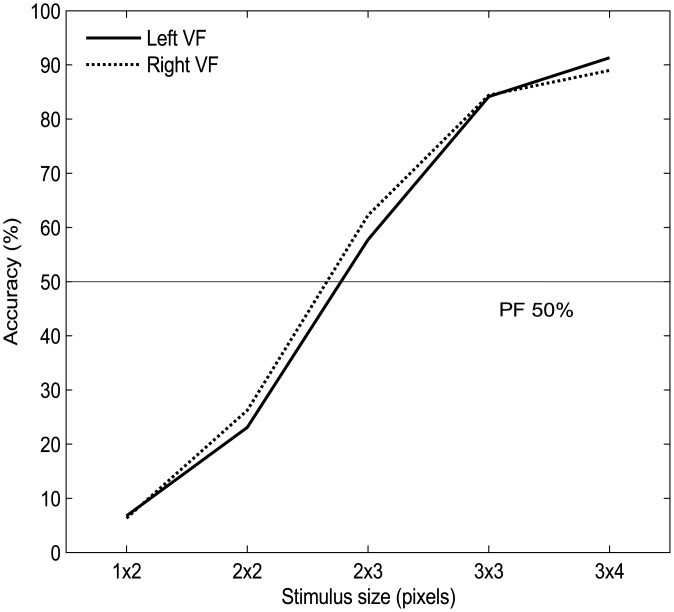
Mean detection accuracy for the LVD task. Separate curves for the left and right VFs are shown, across the 5 stimulus sizes.

Mean accuracy of dot detection is illustrated in Fig 4 per stimulus size and hemispace, revealing no apparent asymmetry between the two visual fields. Averaged across the 5 pixel sizes, stimuli presented on the left were detected with an overall mean accuracy of 52.6% and 53.7% for right-presented stimuli.

**Analysis method 1: D-prime (*d*’):** 88.33% of catch trials were correctly rejected, with 44.29% of the total false positives made towards the left VF and 55.71% to the right (no difference between sides, p>0.05). Mean group-average *d*’ bias (*d’ RVF* minus *d’ LVF)* was 0.022 (collapsed across days). This was not significantly different from zero (one sample t-test, t(49) = 0.69, p = 0.496) hence not revealing a lateralized bias.

**Analysis method 2: Psychometric function fitting (PF 50%):** One participant was excluded from this analysis due to poor performance (18.3% accurate on Day 1, 6.7% on Day 2) which resulted in a very large PF 50% value. The mean (Day 1, Day 2) group-averaged PF 50% for *left*-presented stimuli was 2.81 and 2.72 for *right*-presented stimuli. A repeated measures ANOVA (2 x SIDE, 2 x DAY) showed no PF 50% differences across stimulus presentation location (SIDE: F(1, 48) = 2.037, p = 0.160, ηp^2^ = 0.41) or testing day (DAY: F(1,48) = 2.255, p = 0.140, ηp^2^ = 0.045) and no interaction between the two factors. Accordingly, the mean group-averaged lateralised bias, which amounted to 0.09 (PF 50% *RVF* minus *LVF*) was again not significantly different from zero (one-sample t-test: t(48) = 1.420, p = 0.162).

### Summary of overall task bias

**Fig 5:**
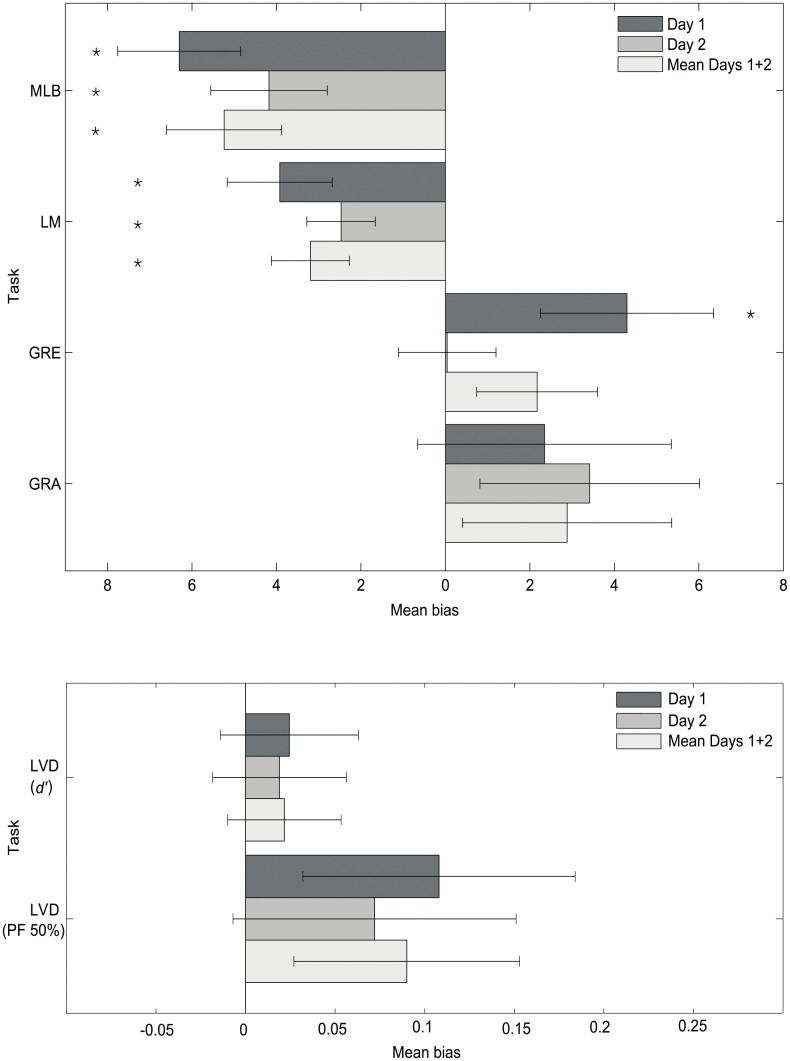
Grand average spatial attention bias for the 5 tasks. Negative and positive values represent leftward and rightward biases respectively. The LM and MLB tasks show significantly leftward biases on both days and the GRE rightward on Day 1 only (± standard error of the mean (SEM)). The LVD task (d’ and PF 50%) is presented separately on the lower axes for clarity, due to smaller bias values. * represents a significant attentional bias compared to zero (p<0.05).

The above group-level results per each of the five tasks are summarized in Fig 5, as well as split by Day 1 and Day 2. One-sample t-tests against zero confirmed an overall significant leftward bias (pseudoneglect) on both days for the MLB task [Day 1: t(49) = -4.330, p<0.001; Day 2: t(49) = -3.026, p = 0.004] and LM task [Day 1: t(49) = -3.158, p = 0.003; Day 2: t(49) = -3.049, p = 0.004]. There was a significant, weak rightward bias for the GRE task on Day 1 [t(49) = 2.098, p = 0.041] but not Day 2. Neither the GRA nor the LVD tasks elicited a lateralised bias on either testing day.

### Intra-task reliability

**Fig 6:**
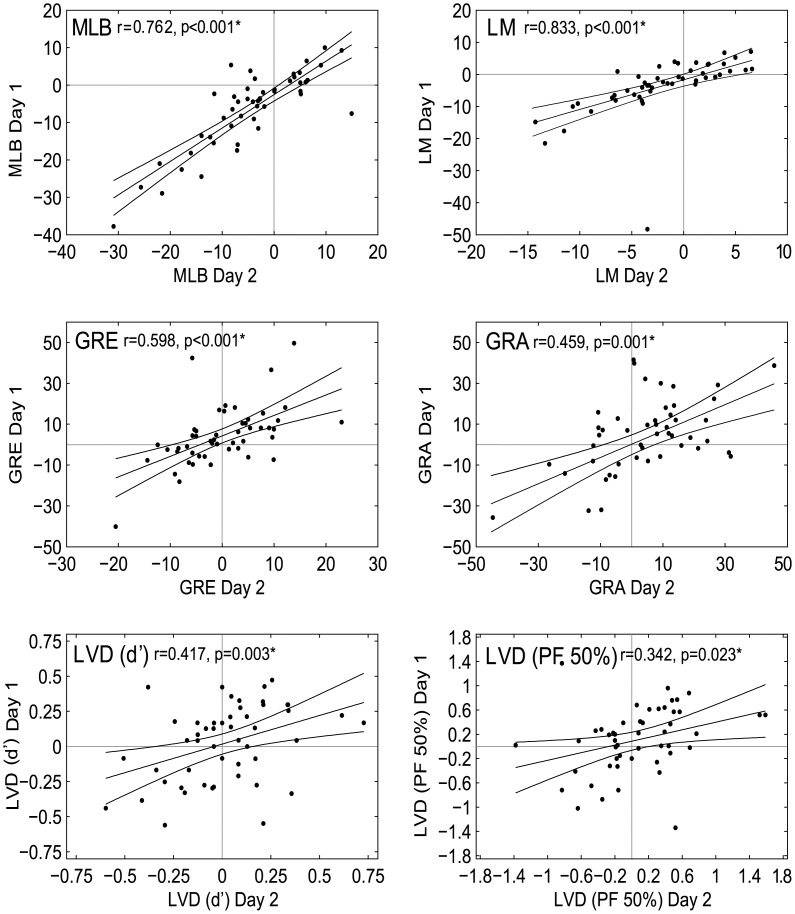
Intra-task correlations. Day 1 vs Day 2 biases are significantly correlated for all 5 tasks (i.e. each task provides a stable measure) over the two testing days (all p-values <0.05). Line of best fit and 95% confidence intervals are marked. * represents a significant correlation at α = 0.05.

A series of Pearson’s *r* correlation tests were used to assess the intra-task test-retest reliability of performance between Day 1 and Day 2 across participants. The biases obtained on all five tasks were correlated across testing days (Fig 6), showing that each measure is a stable indicator of individual spatial attention bias despite some tasks not scoring on an overall bias. Pearson’s *r* of Day 1 vs Day 2 were: [MLB: r = 0.846, p<0.001; LM: r = 0.595, p<0.001; GRE: r = 0.564, p<0.001; GRA: r = 0.560, p<0.001; LVD (*d*’): r = 0.395, p = 0.005; LVD (PF 50%): r = 0.342, p = 0.023].

### Inter-task reliability

Since each of the five tasks provided strongly correlated measures across the two testing days, further analysis was performed on the mean bias across days. Pearson’s *r* correlations on these mean values assessed whether the five tasks elicited comparable measures of spatial attention bias. The correlation coefficients are provided in Table 1.

**Table 1:** Inter-task correlations.

	LM	GRE	GRA	LVD (*d*’)	LVD (PF 50%)
**MLB**	r = 0.267	r = -0.218	r = -0.287	r = -0.182	r = -0.183
	p = 0.06	p = 0.128	p = 0.043*	p = 0.205	p = 0.208
**LM**	—	r = -0.089	r = 0.113	r = -0.147	r = -0.112
		p = 0.537	p = 0.436	p = 0.308	p = 0.445
**GRE**		—	r = 0.161	r = -0.167	r = -0.149
			p = 0.264	p = 0.247	p = 0.305
**GRA**			—	r = 0.047	r = 0.038
				p = 0.744	p = 0.798
**LVD (*d*’)**				—	r = 0.937
					p <0.001**

Correlations performed on the mean task bias (Day 1 and Day 2 averaged).

* significant p-value at α = 0.05 but not significant at the Bonferroni-corrected α = 0.005.

** significant at α = 0.005.

**Fig 7:**
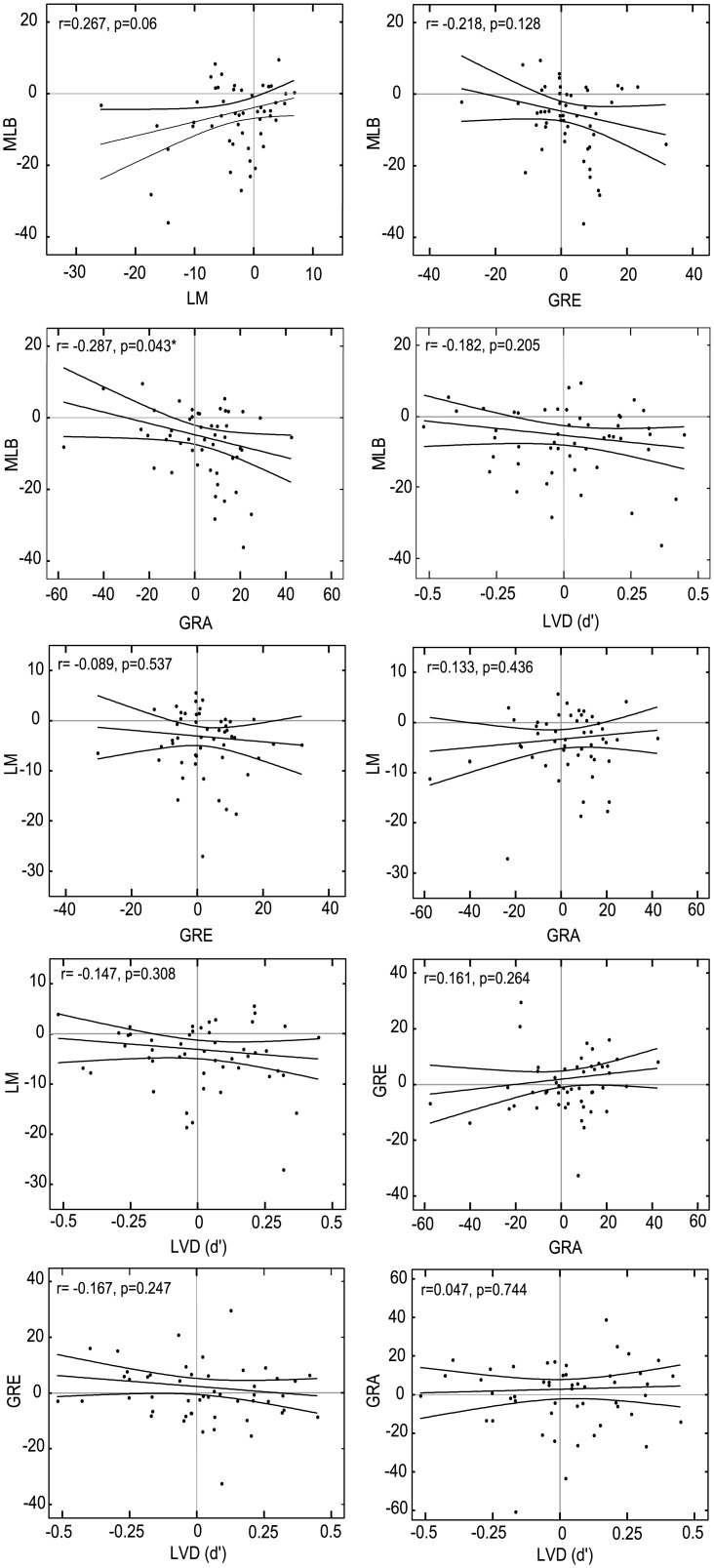
Inter-task correlations. Only the mean task biases (Day 1 and Day 2 averaged) for the MLB and GRA tasks were significantly correlated at α = 0.05 prior to correction, with all other comparisons p>0.05. * represents significant correlation at α = 0.05 but not when Bonferroni corrected to α = 0.005.

Only the MLB and GRA tasks provided significant (yet negative and weakly) correlated mean measures of bias at α = 0.05 that failed to maintain significance when the alpha was Bonferroni corrected for multiple comparisons (Pearson’s *r* = -0.287, p = 0.043, adjusted α = 0.005). No other significant correlations between the five tasks were observed (see also Fig 7).

### Principal component analysis (PCA)

In order to determine whether a smaller number of variables could account for the variance between the tasks, a principal component analysis (PCA) was performed on the correlation matrix using an orthogonal varimax rotation with Kaiser normalisation. Three components with an eigenvalue > 1 were identified which explained 76.15% of the total variance (Table 2). A fourth component with an eigenvalue of 0.65 was forced to assess whether the LVD task loaded onto it and this 4-component model explained 89.1% of the total variance.

**Table 2:** PCA loadings.

	PC1	PC2	PC3	PC4
**Variance explained**	30.6%	24.56%	20.99%	12.95%
**Eigenvalue**	1.53	1.23	1.05	0.65
**MLB**	-0.618	0.544	-0.177	-0.166
**LM**	0.109	0.926	-0.025	-0.052
**GRE**	0.101	-0.060	0.986	-0.093
**GRA**	0.909	0.162	0.053	-0.006
**LVD (d’)**	0.046	-0.082	-0.092	0.988

**Fig 8:**
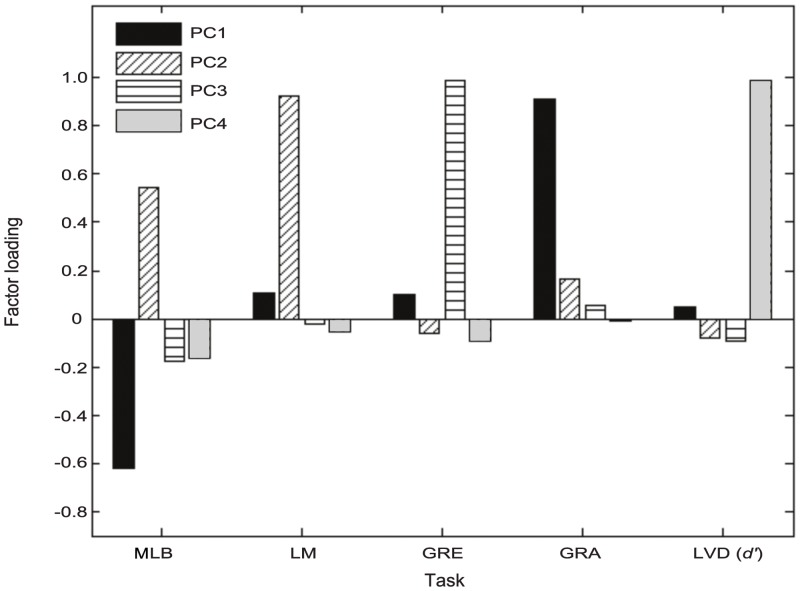
Visualisation of principal component analysis (PCA) loadings.

There was a strongly positive loading for the GRA task on the first principal component (PC1), whilst the MLB task loaded negatively onto this component. Both the LM and MLB tasks loaded together on PC2 and only the GRE task onto PC3. The fourth forced component (PC4) with a lower eigenvalue was found to have a strongly positive loading for the LVD task only (Fig 8).

## Discussion

The results demonstrate that each of the 5 spatial attention tasks assessed here represent consistent and reliable measures of spatial attention asymmetry when administered on different days. Individual spatial biases in each task were reliably replicated between two days of measurements, despite not all tasks showing an overall (grand-average) bias for one direction. Indeed, only two of the five tasks (MLB and LM) elicited a significant stable bias to the left side of space that is consistent with pseudoneglect. A mean *rightward* bias was found in the GRE task on the first day of testing, whereas there was no mean lateralised bias when participants were re-tested. Neither the GRA nor the LVD tasks produced a significant spatial attention bias towards either side of space. Secondly, when the tasks were inter-correlated, only a weak relationship (that did not survive Bonferroni correction) was found between the MLB and GRA tasks, with no other statistically significant relationships observed between any of the other tasks. Importantly, principal component analysis (PCA) identified 4 main components that accounted for 89.1% of the overall variance. The MLB and LM tasks both loaded onto the same component, whereas there was a negative loading relationship between the MLB and GRA tasks on another component. The GRE and LVD tasks were both explained by two further independent factors, indicating that like hemispatial neglect, spatial attention asymmetries in healthy adults involve multiple components, possibly associated with different task demands.

### Intra-task correlations

The stable measures of spatial bias across testing days, as indexed by the test-retest correlations, are broadly consistent with the previous literature [10, 28, 32, 55]. We have extended the study of Nicholls et al. [10] in showing consistency on a range of measures on two different testing days, rather than split-half reliability within a single testing session. Most notably, we failed to find the high performance variability that was reported by Manning et al., [19] for the MLB task: in fact the MLB task provided the strongest intra-task correlation across testing days (r = 0.846). We therefore conclude that the five tasks tested here each index a consistent property of the attention network of each individual that is stable across time.

### Inter-task correlations

We predicted that if pseudoneglect manifests as a result of interhemispheric (left, right) activation differences within a single cortical location, for example the right posterior parietal cortex, that a strong correlational relationship would exist between the five tasks. Although the MLB and GRA tasks proved to hold the closest (negative) correlation between measures of asymmetry relative to the other 3 tasks (r = -0.287, p = 0.043) this did not survive multiple comparison correction. We did not replicate the previously reported (although again weak) correlations between the MLB and GRE tasks [38, 46] nor the correlation between the (procedurally similar) GRE and GRA tasks previously described by Niemeier et al., [48]. Conversely, we replicated the previous findings of Luh [9], Nicholls et al., [10] and Heber et al., [45] showing a lack of inter-task relationship between measures of pseudoneglect and have demonstrated that tasks used in the current spatial attention literature exhibit the same lack of strong inter-task correlation that was demonstrated almost twenty years ago. It should be noted that with our sample size of 50 individuals, the within-subjects design, and the stability of all 5 measures over time as indexed by the intra-task correlations, it is most likely that the experiment provided adequate statistical power to have highlighted any relationships between tasks, should they exist.

### Differences in measures of spatial bias

PCA identified four separate components which explained 89.1% of the total correlation matrix variance. Although the biases we report for the MLB and LM tasks were only very weakly correlated (r = 0.267, p = 0.06), the co-loading of these two tasks on to one single component (PC2) supports the behavioural [9, 11, 18, 22] and fMRI [12, 35] evidence that a similar pattern of neural activation underpins the completion of both of these tasks. We have chosen to label PC2 as representative of a “global size judgement” task demand, since the MLB and LM tasks both involve an assessment of the midpoint location along a single, continuous horizontal line. This finding fits well with Verdon et al., [8] who found that MLB task performance loaded onto a “perceptive/visuo-spatial” component in patients with hemispatial neglect, and in which impairment on this task was closely associated with damage to the right inferior parietal lobe. Moreover, we found that only the MLB and LM tasks produced a significant mean leftward bias in both testing sessions. Tasks involving global size judgement may therefore be more sensitive to detecting asymmetries in spatial attention than tasks which require visual assessment of other stimulus features.

The relative independence of the GRE loading on PC3 (which we have labelled “luminance judgement”) is perhaps surprising given that the GRE and GRA tasks are procedurally similar, with both requiring a comparison of the relative area containing target stimulus features between two parallel horizontal bars. Yet since they both load strongly onto different PCA components we are confident that this highlights differences in task demands (i.e. the focus on luminance versus spatial frequency).

Similar to the GRE, the LVD task also loaded independently on to PC4 (although note that the eigenvalue was <1), which we have labelled “stimulus detection”. Contrary to our previous findings [55] we found no grand-average visual detection sensitivity bias towards the left hemispace. Given that the two methods of analysis (*d*’ and PF 50%) produced highly correlated measures of LVD bias, we conclude that this lack of bias was likely due to procedural differences rather than an artefact of analysis. Most notably, previous studies using the LVD [53–57] have titrated the stimuli for each individual and presented 2 peri-threshold stimuli to ensure that the task was of equable difficulty across participants. Here instead, we chose to present a standard set of 5 stimulus sizes which may have been less sensitive to the detection of spatial bias due to reduced (or *increased* for some individuals) task difficulty that may have differentially influenced the balance of activity within the spatial attention network. Given that the LVD did not correlate with any other task and loaded independently onto PC4, we can conclude that the LVD as presented here relies upon attention mechanisms that are procedurally distinct from the other tasks tested.

The *inverse* relationship between the MLB and GRA loadings on PC1 is more complex to explain. For the MLB task, participants assessed the *global* properties of the stimulus by visually scanning the entire line length and determining the midpoint location over a relatively long trial duration (maximum = 6 seconds). Conversely, the GRA involved fast stimulus presentation (150ms) during which participants were directed to assess the quantity of fine-grained, *local* stimulus features (i.e. the number of “thin stripes”) in the absence of visual scanning. Therefore, labelling PC1 as representative of “*local vs global*” processing fits the divergent task demands of MLB and GRA presented here. This interpretation also aligns with models of spatial attention which posit that tasks involving fine-grained perceptual judgements (the processing of “*local*” stimulus features and HiSF gratings) are processed using LH resources, whereas the RH supports decisions regarding larger scale, “*global*” stimulus features and LoSF gratings [63–64]. The model would therefore predict a leftward spatial bias when LoSF gratings are assessed and a rightward bias in response to HiSF targets.

More specifically, and contrary to this model, Niemeier et al., [48] found a leftward attentional bias for HiSF targets and a rightward bias for LoSF, possibly due to the comparatively high salience of HiSF relative to LoSF that preferentially activates the RH [51]. We failed to find a spatial bias in our sample even though participants were directed to the HiSF features. However, Niemeier et al. [52] have also shown that the leftward bias in the GRA task is more pronounced when the HiSF gratings span a spatial frequency range of 0.6–2 cycles per degree (cpd). Thus our stimulus choice of HiSF 4.07 cpd may have proved less sensitive to detecting an underlying spatial asymmetry, although a weak correlation between the GRE and GRA using similar stimulus parameters (stimulus G4 in [48]) was still noted. So if we have correctly labelled PC1 as being representative of *global vs local* feature processing, and participants show an inverse PCA loading relationship between the MLB and GRA tasks, then the results may indeed align with the RH-global LH-local model.

### Pseudoneglect as a multi-component phenomenon

In line with the current conceptualisation of hemispatial neglect as a multi-component disorder, we would argue that pseudoneglect may also be conceptualised as multi-faceted. Patients with neglect demonstrate large individual performance variability on tasks that involve different task demands (e.g. text reading, object cancellation, line bisection) and are dependent on the location of their lesions [3, 8, 65]. Correspondingly, we have shown here that the direction and magnitude of spatial bias in healthy young adults is strongly task-dependent and therefore likely to be related to partially-overlapping regions of the brain which are responsible for the completion of each task. Our results demonstrate that an individual may overestimate the size of the left hemispace as measured by MLB or LM tasks, however it does not follow that they will also exhibit a strong leftward overestimation of luminance or spatial frequency on the left, nor show increased stimulus discrimination sensitivity to this side.

These results fit well with the interhemispheric competition model of spatial attention [66–67], which posits that attentional asymmetries manifest as a result of the relative differences in activation between the left and right cerebral hemispheres (LH/RH). A larger net RH activation biases attention towards the left side of space (resulting in an over-estimation of the stimulus features on this side) and LH activity results in a preference for the right. Alternatively, this variation in bias may reflect a more complex interaction between dorsal (*endogenous*) and ventral (*exogenous*) attention networks [68–69] that is mediated by variations in task demands. If tasks differentially activate areas within the RH and LH then this would predict the inconsistency of spatial bias as displayed here.

### Potential effects of task difficulty and viewing distance

The influence of task difficulty on hemispatial neglect in patients is well documented, with the extent of inattention towards the left hemispace often accentuated as task difficulty increases ([70–74] but see also [75]). Similarly in the non-clinical population, a greater overall attentional engagement with the task might be expected as a function of increased task difficulty. This may actively engage the right hemisphere leading to a leftward bias, or in some cases *deplete* RH functions leading to rightward shift in attention, such as observed with prolonged time-on-task, reduced arousal or increased perceptual load [27, 31, 76–83]. Aligned with this, we have previously reported that an increase in task difficulty (as indexed by a larger psychometric function curve width for short compared to long LM task lines) is accompanied by a rightward shift in PSE [29]. However, Niemeier et al., [48] found no influence of stimulus presentation time (where a shorter presentation time was found to be more difficult as indexed by psychometric function slopes) on the direction of the bias in the GRA task: a leftward bias was observed for both short and long durations.

As a final consideration, the viewing distance of 0.7m here was intended to represent a compromise between the wide range of distances reported in the previous studies on which these tasks were based (approximately 0.3m for paper-and-pencil manual line bisection studies, increasing to 1.0m for some landmark task presentations [27–28]). It has been previously demonstrated that viewing distance can influence the spatial bias obtained in these tasks. Viewing in near space tends to elicit a leftward bias in both landmark and line bisection tasks, with a reduced bias (or slightly rightward) when viewed in far space [14, 84–93]. It is conceivable that the overall lack of spatial bias observed for the GRA and LVD tasks (and the GRE on Day 2) shown here was generated partially due to this aspect of our experimental design. However, given that all 5 tasks were presented at the same viewing distance, we expected to see stronger inter-task correlations than we ultimately observed. Therefore, regardless of whether the variations in perceptual asymmetry we observed were related to task-dependent differences in cortical activation, task difficulty, viewing distance or otherwise, we urge caution in assuming an equivalency of tasks when designing spatial attention experiments.

## Supporting Information

S1 Data Spreadsheet containing individual participant data. Columns C-F: Alertness scores. Columns H-S: Spatial bias scores. Columns U-AD: Curve width values. Columns AF-AN: Mean spatial bias scores for each MLB stimulus position.
(XLSX)
