# Correction: Intra- and Inter-Task Reliability of Spatial Attention Measures in Pseudoneglect

**DOI:** 10.1371/journal.pone.0205269

**Published:** 2018-10-02

**Authors:** Gemma Learmonth, Aodhan Gallagher, Jamie Gibson, Gregor Thut, Monika Harvey

There is an error in the analysis pipeline for the greyscales (GRE) and gratingscales (GRA) tasks. This has led to all spatial bias scores (PSEs) for the greyscales and gratingscales tasks having inverted signs: all positive values (i.e. rightward biases) should be negative (i.e. leftward biases), and vice versa. The spatial bias values per se are not affected. All other tasks are unaffected and this does not change the main message of the article (i.e. significant intra-task correlations across 2 testing days, but no inter-task relationships between the 5 tasks). This single error in the processing pipeline has led to the following errors throughout the article:

There is an error in the Methods section under the subheading “LM, GRE and GRA tasks.” The second sentence should read: Accuracy for each of the 17 stimulus asymmetries was converted into a percentage of trials where the subject perceived the stimulus to be either **longer** (LM) / darker (GRE) / have more “thin stripes” (GRA) on the **right** side of space.

There are several errors in the Results section. The third sentence under the subheading “Summary of overall task bias” should read: There was a significant, weak **leftward** bias for the GRE task on Day 1 [t(49) = 2.098, p = 0.041] but not Day 2. The first sentence of the second paragraph under the subheading “Inter-task reliability” should read: Only the MLB and GRA tasks provided significant **(yet weakly)** correlated mean measures of bias at α = 0.05 that failed to maintain significance when the alpha was Bonferroni corrected for multiple comparisons (Pearson’s r = -0.287, p = 0.043, adjusted α = 0.005). The first sentence of the second paragraph under the subheading “Principal component analysis (PCA)” should read: There **were** strongly positive loading**s** for the GRA **and MLB** tasks on the first principal component (PC1).

There are several errors in the Discussion section. The fourth sentence of the first paragraph should read: A mean **leftward** bias was found in the GRE task on the first day of testing, whereas there was no mean lateralised bias when participants were re-tested. The second sentence under the subheading “Inter-task correlations” should read: Although the MLB and GRA tasks proved to hold the closest correlation between measures of asymmetry relative to the other 3 tasks (r = -0.287, p = 0.043) this did not survive multiple comparison correction.

In Fig 3, the y-axis label is incorrect. The authors have provided a corrected version here.

**Fig 3:**
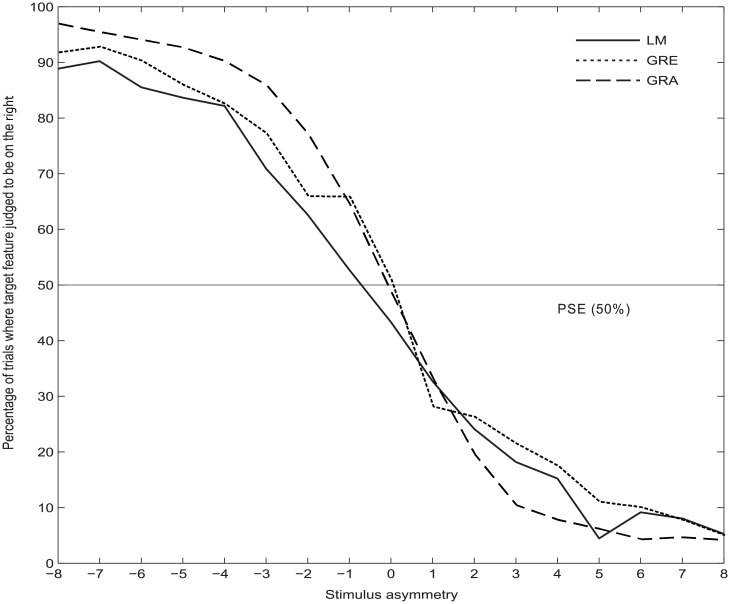
Mean psychometric function curves for the LM, GRE and GRA tasks. The asymmetry of the presented stimulus is shown on the x-axis, where 0 = “both sides equal length” (LM) or “both bars equal darkness/thin stripes” (GRE/GRA respectively). Negative asymmetry values represent trials where the target feature is located on the left side and positive values on the right side. One unit on the x-axis equates to 3 pixels (0.07°) for the LM task, 10 pixels (0.24°) for GRE and 12 pixels (0.29°) for GRA.

In Fig 5, the mean PSE bars for the GRE and GRA tasks (Day 1, Day 2 and Mean Days 1+2) are incorrect. Additionally, the second sentence of the caption is incorrect. Please see the corrected Fig 5 and caption here.

**Fig 5:**
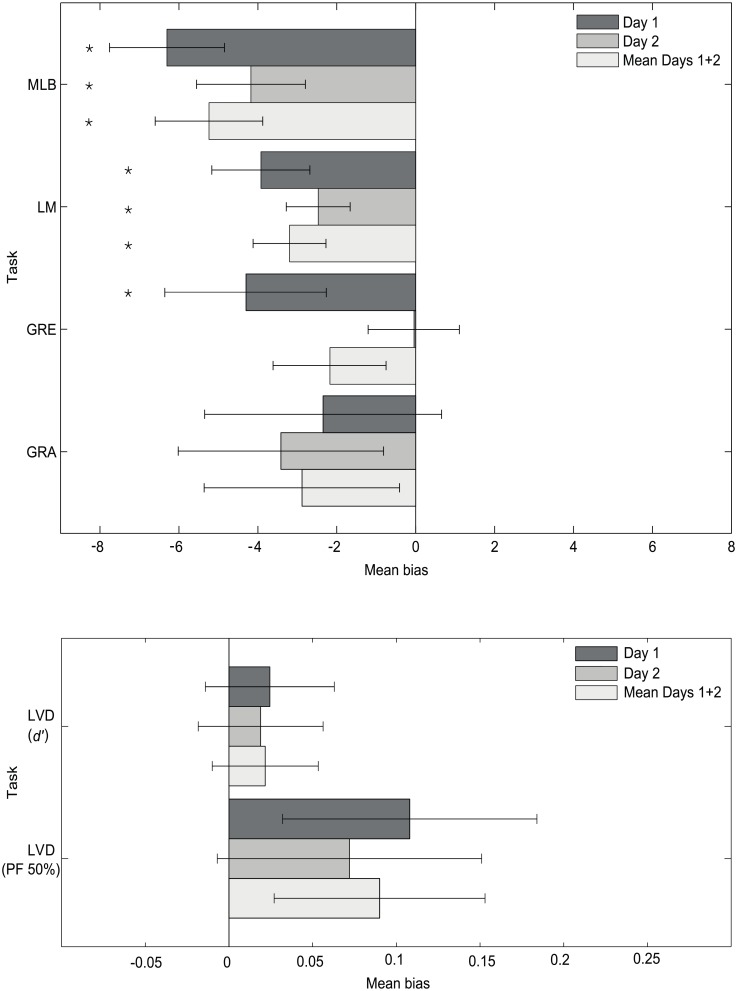
Grand average spatial attention bias for the 5 tasks. Negative and positive values represent leftward and rightward biases respectively. The LM and MLB tasks show significantly leftward biases on both days and the GRE **leftward** on Day 1 only (± standard error of the mean (SEM)). The LVD task (d’ and PF 50%) is presented separately on the lower axes for clarity, due to smaller bias values. * represents a significant attentional bias compared to zero (p<0.05).

In Fig 6, the correlation plots for the GRE and GRA tasks are incorrect. The authors have provided a corrected version here.

**Fig 6:**
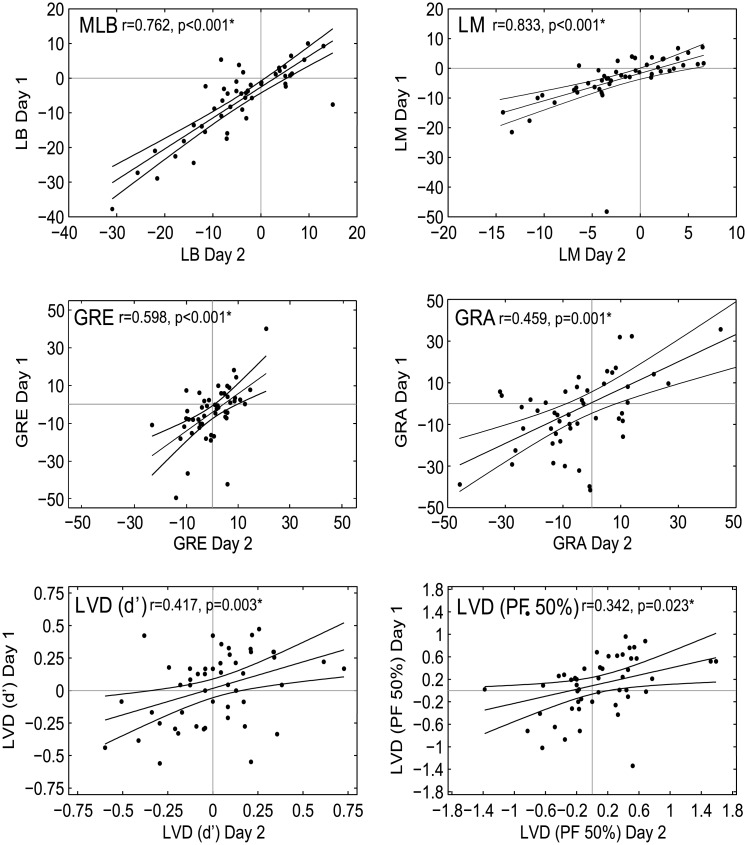
Intra-task correlations. Day 1 vs Day 2 biases are significantly correlated for all 5 tasks (i.e. each task provides a stable measure) over the two testing days (all p-values <0.05). Line of best fit and 95% confidence intervals are marked. * represents a significant correlation at α = 0.05.

In Fig 7, the direction of the relationship for the GRE and GRA are incorrect. Please see the corrected version here.

**Fig 7:**
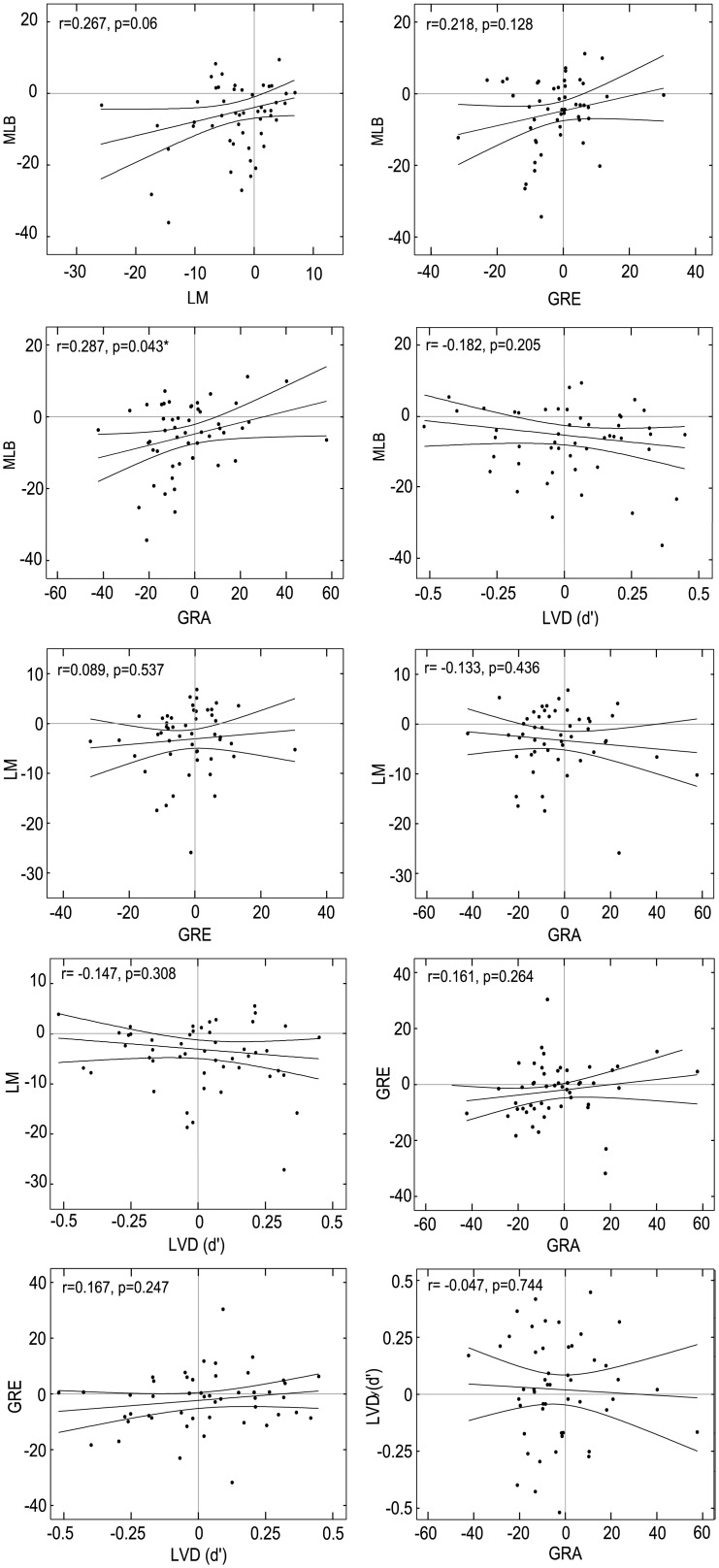
Inter-task correlations. Only the mean task biases (Day 1 and Day 2 averaged) for the MLB and GRA tasks were significantly correlated at α = 0.05 prior to correction, with all other comparisons p>0.05. * represents significant correlation at α = 0.05 but not when Bonferroni corrected to α = 0.005.

In Fig 8, the PCA values are incorrect. Please see the corrected version here.

**Fig 8:**
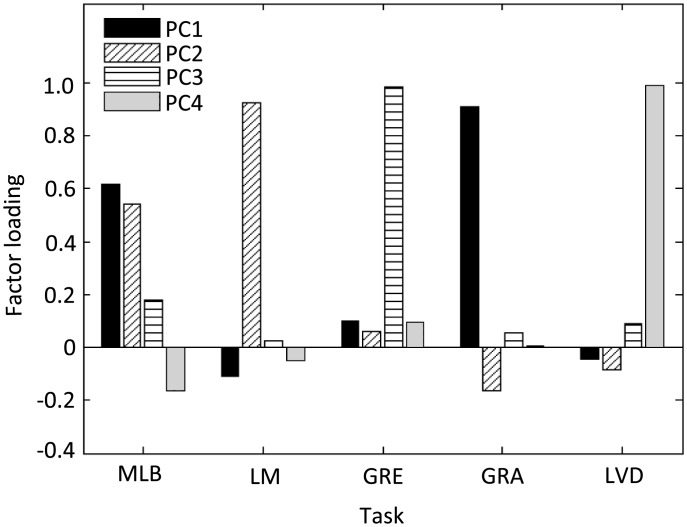
Visualisation of principal component analysis (PCA) loadings.

There is an error in Table 1. Data of the GRE and GRA tasks have been amended (multiplication of all values by -1). Please see the corrected Table 1 here.

**Table 1:** Inter-task correlations.

	LM	GRE	GRA	LVD (*d’*)	LVD (PF 50%)
MLB	r = 0.267	r = 0.218	r = 0.287	r = -0.182	r = -0.183
	p = 0.06	p = 0.128	p = 0.043*	p = 0.205	p = 0.208
LM	--	r = 0.089	r = -0.113	r = -0.147	r = -0.112
		p = 0.537	p = 0.436	p = 0.308	p = 0.445
GRE		--	r = 0.161	r = 0.167	r = 0.149
			p = 0.264	p = 0.247	p = 0.305
GRA			--	r = -0.047	r = -0.038
				p = 0.744	p = 0.798
LVD (*d’*)				--	r = 0.937
					p <0.001**

There are several errors in the PCA loadings for Table 2. Please see the corrected version here.

**Table 2:** PCA loadings.

	PC1	PC2	PC3	PC4
**Variance explained**	30.6%	24.56%	20.99%	12.95%
**Eigenvalue**	1.53	1.23	1.05	0.65
**MLB**	0.618	0.544	0.177	-0.166
**LM**	-0.109	0.926	0.025	-0.052
**GRE**	0.101	0.060	0.986	0.093
**GRA**	0.909	-0.162	0.053	0.006
**LVD (d’)**	-0.046	-0.082	0.092	0.988
